# The Association Between Sociability and COVID-19 Pandemic Stress

**DOI:** 10.3389/fpsyg.2022.828076

**Published:** 2022-02-22

**Authors:** Peihao Luo, Matthew L. LaPalme, Christina Cipriano, Marc A. Brackett

**Affiliations:** Yale Center for Emotional Intelligence, Yale Child Study Center, Yale School of Medicine, Yale University, New Haven, CT, United States

**Keywords:** COVID-19, stress, social isolation, sociability, social interaction, social support, minorities, health

## Abstract

The COVID-19 pandemic threatened our physical health, alongside our mental and social wellbeing. Social distancing requirements, which are necessary to mitigate the spread of COVID-19, increased social isolation by limiting social interactions that are an essential part of human wellbeing. In this study, we examined the stress caused by COVID-19 early on in the pandemic through the lens of sociability among a large sample of preservice educators (*N* = 2,183). We found that individuals who have higher sociability (including deriving joy from social interactions and using social support to manage emotions) experienced greater COVID-19 stress. This study also contributed to prior literature which has sought to relate pandemic-related stress to demographic group differences. We found no significant relationship between demographic membership (gender, race, and sexual orientation) and COVID-19 stress. This study is among the first, however, to demonstrate that vulnerability to pandemic stress varies as a function of sociability. Implications of these findings and ways people can better cope with pandemic isolation are discussed.

## Introduction

The COVID-19 pandemic dramatically affected people’s lives, with adverse physical and mental health impacts (Kumar and Nayar, 2020; Restubog et al., 2020; O’Connor et al., 2021). At the height of the pandemic, country-level estimates of pandemic stress were as high as 40% (Montano and Acebes, 2020), and in the U.S. more than 40% of people reported adverse mental health experiences (Czeisler et al., 2020). The impact of this stress was significant, and recent studies suggest that COVID-19 stress may be a risk factor for more severe health issues like PTSD (Di Crosta et al., 2020) Recent meta-analysis studies showed that the global prevalence estimate for stress during COVID-19 was 36.5%, and globally, nearly one-quarter of the population experienced posttraumatic stress symptoms as a result of the pandemic (Cooke et al., 2020; Nochaiwong et al., 2021). Emerging research has examined demographic differences in COVID-19 stress (e.g., Kowal et al., 2020; Montano and Acebes, 2020; Yalçın et al., 2021); however, there is little research currently available on how COVID-19 stress affects individuals differently based on their sociability. Because social distancing requirements due to the pandemic have substantially altered social interaction patterns, differences in sociability may be an important bellwether for determining who may be more susceptible to experiencing pandemic stress.

The current study explored whether individual differences in sociability is related to experiencing different levels of COVID-19-related stress early on in the pandemic. Our focus was on the stress related to the pandemic, rather than having or contracting COVID-19. The negative effects of the COVID-19 pandemic can be long lasting and determining who is most vulnerable to pandemic-related stress would be beneficial for prevention and for supporting groups that need the most help and guidance. We examined the extent to which individuals who derived joy from social interactions or seek social support as a emotion regulation strategy appraised the COVID-19 pandemic as a significant source of stress.

### COVID-19 Pandemic and Social Isolation

To reduce the spread of COVID-19, many countries instituted social distancing policies restricting individuals from social interaction to curtail the spread of the virus (Matrajt and Leung, 2020; World Health Organization, 2020a). While these measures are essential to prevent the virus from spreading, limitations on social interaction could cause unintended psychological harm (Cudjoe and Kotwal, 2020; Von Mohr et al., 2021). Imposed isolation can lead to increased negative emotions, including boredom, anger, and loneliness (Montano and Acebes, 2020). The WHO has warned that self-isolation and social distancing increase depression, anxiety, and stress (World Health Organization, 2020b), and emerging research has shown that experiencing long-term quarantine is associated with increased rates of depression and PTSD (Montano and Acebes, 2020). The amount of time spent in social isolation has also been found to be associated with increased stress during COVID-19, and people with higher levels of COVID-19 stress tend to view social isolation as more distressing (Taylor et al., 2020b), creating a harmful downward spiral of isolation and stress.

### Social Isolation and Individual Differences in Sociability

Some individuals may be more (or less) vulnerable to the social isolation caused by quarantine requirements of COVID-19. For example, Taylor et al. (2020b), found that people who lived alone before the pandemic experienced lower COVID-19 stress. This indicates that people may appraise social isolation differently based on their sociability level. Appraisal theory posits that stress is generated by evaluations of events or situations (Roseman and Smith, 2001); that is, it is the interpretation of the event or situation that drives one’s experience. Thus, individual experience can vary greatly when facing the same event (e.g., Smith and Ellsworth, 1987; Shaver et al., 1988; Smith and Pope, 1992). Regarding social interaction, some people may view social interaction as a source of joy, while others may appraise social interaction as either neutral or as a stressful resource drain.

Although social interactions are important to the maintenance of mental and physical health (Cacioppo et al., 2011), some people prefer solitude, which researchers distinguish from loneliness. Loneliness involves an appraisal of isolation as a negative emotional experience (Lay et al., 2020). Though solitude is sometimes viewed as a sign of isolation and unsociability, research shows that solitude can be a volitional preference that can enhance wellbeing for those who prefer it (Lay et al., 2020). Individuals who intentionally seek solitude are less likely to feel lonely from social isolation (Lay et al., 2020).

Conversely, isolation due to COVID-19 may be more stressful to those who are high on *sociability* (McCrae and Costa, 1987). Individuals higher in sociability prefer to talk or interact with others more frequently and enjoy such interactions (Cheek and Buss, 1981; McCrae and Costa, 1987). A similar term is *need for affiliation*, which can be described as the trait desire for social contact and belonging (Wiesenfeld et al., 2001; Veroff and Veroff, 2016). Compared to those who prefer solitude, those who are high in sociability and need for affiliation experience and derive relatively more joy from interacting with family and friends (Hill, 1987; Eisenberg et al., 1995). However, this can also make them vulnerable to the effects of social isolation. It is possible that as a result of the COVID-19 pandemic, new norms of social isolation (e.g., quarantine and social distancing) may be appraised by individuals who enjoy social interaction as particularly stressful and harmful. Research shows that the effect of social isolation on mental health can compound for these individuals over time (Pancani et al., 2021).

In this study, we hypothesize that individuals high on sociability who appraise social interaction as a source of joy (for simplicity, we refer to social interaction as a source of joy as *social joy*) experience more COVID-19 stress.

### Social Support and Individual Differences in Sociability

The most common strategies used to cope with COVID-19 stress are distraction, active coping, and seeking emotional or social support (Park et al., 2020). Seeking social support is one of the most common ways to cope with stressors (Carver et al., 1989; Yalçın et al., 2021). Seeking social support can include the experience of seeking love and appreciation through one’s social network (e.g., from family or friends; Carver et al., 1989; Wills, 1991). Research has shown that social support can be essential for mental and physical health when coping with stressful life events (Mortenson, 2009; Lay et al., 2020). It is thought to be beneficial because it is a deactivating strategy that reduces the experience of stress while enhancing wellbeing (Taylor, 2007). Social support has even been shown to help patients to recover from illness (Taylor et al., 2004).

Though seeking social support can be useful in many situations, some may prefer to seek more social support while others less social support. Sensitive interaction systems theory (SIST) explores the process of seeking social support. According to SIST, people make decisions to seek social support based on many variables, including the threat to self-esteem, likelihood of rejection, and perceived cost of seeking social support (Barbee and Cunningham, 1995). People with low self-esteem or people that fear rejection, for example, are more likely to cope alone instead of using social interaction (Mortenson, 2009). Likewise, prior research has shown gender differences with men less likely to seek social support than women (Carver et al., 1989). Individuals high on sociability also differ in how they regulate and cope with difficult feelings—tending to prefer to seek more social support and tend to use fewer avoidant strategies (Hill, 1987; Vollrath et al., 1995). Taken together, individuals may benefit more (or less) from social support and differentially seek social support depending on their appraisals (Mortenson, 2009; Feeney and Collins, 2015). A recent study showed that during COVID-19, those who have higher preference on touch for affect regulation (TAR; a way of seeking emotional support from others) but experienced less affectionate touch reported more psychological distress that those who have lower TAR (Burleson et al., 2021). Given that COVID-19 quarantine restrictions limit the ability of individuals to engage in social interaction, in this study we hypothesized that individuals high on sociability who prefer social support as a regulatory strategy (for simplicity, we refer to social support as a regulatory strategy as *social regulation*) would appraise COVID-19 as more stressful.

### Demographic Differences on COVID-19 Stress

Demographic characteristics, such as race, age, gender, and sexuality, may interact and affect the experience of stress due to COVID-19 (Montano and Acebes, 2020; Taylor et al., 2020b; Rosi et al., 2021). To date, findings relating demographic variables and COVID are mixed. For example, although Taylor et al. (2020b) found white individuals reported lower stress levels during the pandemic compared to their African American and Asian peers, Ponnock et al. (2021) found that African American educators reported less stress than White educators. Similarly, the evidence for differences in stress levels by gender is mixed, with no significant difference between men and women in COVID-19 stress in some studies (Montano and Acebes, 2020; Szabo et al., 2020; Yan et al., 2021), and women reporting to be more vulnerable than men to COVID-19-related mental health issues including stress in other studies (Ahuja et al., 2020; Mazza et al., 2020; Yalçın et al., 2021). Further, differences across age group experiences of stress have also been observed. Individuals from 41 to 50 years old scored the lowest on COVID-19 stress compared to other younger adult groups or adolescents (Montano and Acebes, 2020; *cf.* Rosi et al., 2021), and students and young adults are more vulnerable to COVID-19 stress (Balsamo and Carlucci, 2020), especially those under 35 (Huang and Zhao, 2020). To address these issues, in the present study we further seek to unpack the potential impact of demographic characteristics on experiences of COVID-19 stress.

In summary, the current study aims to test the following hypothesis:

*Hypothesis 1*: Individuals high on sociability that appraise social interaction as a source of joy (higher in social joy) will experience more COVID-19 stress.

*Hypothesis 2*: Individuals high on sociability who prefer social support as a regulatory strategy (higher in social regulation) will experience more COVID-19 stress.

Additionally, we also examined whether there were any differences on COVID-19 stress across demographic groups.

Research Question 1: Are there demographic group differences on COVID-19 stress?

## Materials and Methods

To explore our hypotheses, we used an open-ended survey study to capture COVID-19 stress, social joy, and social regulation among educators. We chose to examine these phenomena in a large sample of early-career educators because prior studies have noted the severe emotional impact the pandemic has had on educators, with anxiety by far being the most frequently mentioned emotion among educators during the pandemic (Brackett and Cipriano, 2020). We collected these data during the pandemic “surge” of the Summer of 2020 in the U.S. when the most stringent social distancing requirements were in effect.

We operationalized COVID-19 stress as the extent to which participants wrote in COVID-19-related words as stressors (e.g., writing “COVID-19” or “the pandemic” as one of their top sources of stress). Furthermore, we operationalized sociability as the extent to which participants wrote in social-related words as their sources of joy (e.g., writing “my husband,” or “my son,” or “meeting new people” as one of their top sources of joy) and regulation strategies (e.g., writing “talking to my partner,” or “conversation with friends/family,” or “spending time with loved ones” as one of their most helpful social emotion regulation strategies). This open-ended approach allowed us to capture the nuances of potential stressors during the COVID-19 pandemic as well as how the pandemic might interfere with social joy and social regulation.

### Participants and Procedure

All participants were preservice educators participating in a teacher training program in summer 2020. Data were collected from June 26^th^ to July 3^rd^. A total of 2,183 individuals completed the survey and were included in the analysis. The sample was on average 24.9 years old (*SD* = 6.0), 75.1% of whom were female with 2.0% identifying as other or not indicating their gender identity. The sample identified as 16.1% African American, 7.4% Asian, 16.4% Hispanic, 8.1% Multiracial, 0.5% Native/Indigenous, and 48.6% White. For sexual orientation, the participants identified themselves as 8.3% bisexual, 2.7% gay, 1.6% lesbian, 3.6% queer, and 69.0% straight; 14.8% choose not to answer or identified as questioning or other.

### Measures

The measures used in this study were part of a larger project focusing on the wellbeing of preservice teachers in Teach for America.

#### Open-Ended Questions

Participants were asked to respond to three open-ended questions, including their: (1) top three sources of stress and anxiety, (2) top three sources of joy, and (3) top three strategies they used to regulate their emotions. The specific questions can be found in Table 1. While psychologists differentiate stress and anxiety (stress being an appraisal of demands exceeding resources and anxiety being an appraisal about uncertainty or worry about the future; Bakker and Demerouti, 2007; Grupe and Nitschke, 2013), research has shown that individuals use these terms interchangeably to describe their experiences. For example, Moeller et al. (2020) found that “stressed” and “anxious” were among the most frequent mentioned emotion words by students, and Ivcevic et al. (2021) reported both stressed and anxious being mentioned frequently by employees simultaneously. Likewise, Floman et al. (2021) found that stress and anxiety frequently co-occur as experiences. In addition, one of the core characteristics of COVID-19 stress is worry or uncertainty about the future (e.g., worry about of getting infected or, facing financial difficulties, or job-related uncertainty) which touches on the core appraisal of anxiety. Indeed, prior research has shown that viral pandemic-related stress is very closely tied to appraisals of uncertainty and feelings of anxiety (see Taha et al., 2014 for empirical studies related to H1N1 stress).

**Table 1:** Open-ended questions.

Open-ended Questions
1. Please reflect on your stress and anxiety over the past few weeks. What are the **top 3** causes of your stress and anxiety? Please list them below.
2. Even in trying times, there can be moments where we experience joy. In the past few weeks, what are the **top 3** things that have brought you **joy**? Please list them below.
3. Please take a moment and reflect on how you are managing your emotions during this difficult time. Over the past few weeks, what 3 strategies/approaches have you found most helpful for managing **your own emotions**? Please list them below.

To fully capture the experience of stress due to the COVID-19 pandemic, we chose to ask about both stress and anxiety in our measure. Each question had three response boxes, one for each of the top three categories. Text from our open-ended data were analyzed using Linguistic Inquiry and Word Count (LIWC; Pennebaker et al., 2015). Prior research has used LIWC to examine personality differences related to social interaction, such as extraversion (e.g., Tausczik and Pennebaker, 2010). For example, Pennebaker and King (1999) found that extraversion is correlated with using social words. Furthermore, more recent studies have also demonstrated that language use correlates with both self-perception and other perception of sociable traits (Mehl et al., 2006). Prior studies have demonstrated that individual difference in linguistic style captured by LIWC is consistent and reliable (Pennebaker and King, 1999; Pennebaker et al., 2003). Below, we describe the specific coding procedures and dictionaries used to define social joy, social regulation, and COVID-19 stress.

### Sociability: Social Joy and Social Regulation

We analyzed both the sources of joy and the emotion regulation strategy questions using the “social” categories from LIWC 2015s default dictionary. These social categories include words related to family (e.g., aunt and baby), friends (e.g., ally and crew), female- or male-related (e.g., girl and boy), and other social-related words (e.g., help and talk). Any social-related terms (e.g., family, friend, and team) are coded by software to indicate the level of sociability. For example, one participant wrote in, “meeting new people” as a source of joy, and this would be coded as higher on appraising social interactions as joyful (social joy). Likewise, one participant wrote, “Talking with my partner” in response to one of the emotion regulation questions, this would indicate stronger preference for social regulation. By using open-ended response coding, our study captures many potential sources of social joy and social regulation.

LIWC 2015 scores the social coding category based on the percentage of key social words presented in the open-ended responses (descriptive statistics are shown below in results). Thus, sociability was operationalized as (1) the percentage of social words written in response to the source of joy prompt and (2) the percentage of social words written in response to the emotion regulation prompt. Here higher percentages mean higher sociability.

#### COVID-19 Stress

We used the same open-ended coding approach to measure COVID-19 stress. The first and second author created a new LIWC 2015 dictionary to capture words related to COVID-19 stress. Each author generated a list of words about COVID-19 independently, met to discuss differences, and reached agreement on a final version of the COVID-19 stress dictionary used for this study. This dictionary included the following words: COVID^*^, pandemic, virus^*^, corona^*^, lockdown, quarantin^*^, sick, ill, health (^*^ indicate word stems, the coding is not case sensitive). For example, one participant wrote in, “COVID-19” as a source of stress, this would indicate more COVID-19 stress. Thus, we operationalized COVID-19 stress as the percentage of COVID-19-related words written in response to the top stressor prompt, and higher percentage meant higher perceived COVID-19 stress.

Because we only asked for the top three stressors, our open-ended measure tended to capture acute COVID-19 stress rather than mild cases of COVID-19 stress.

**Figure 1:**
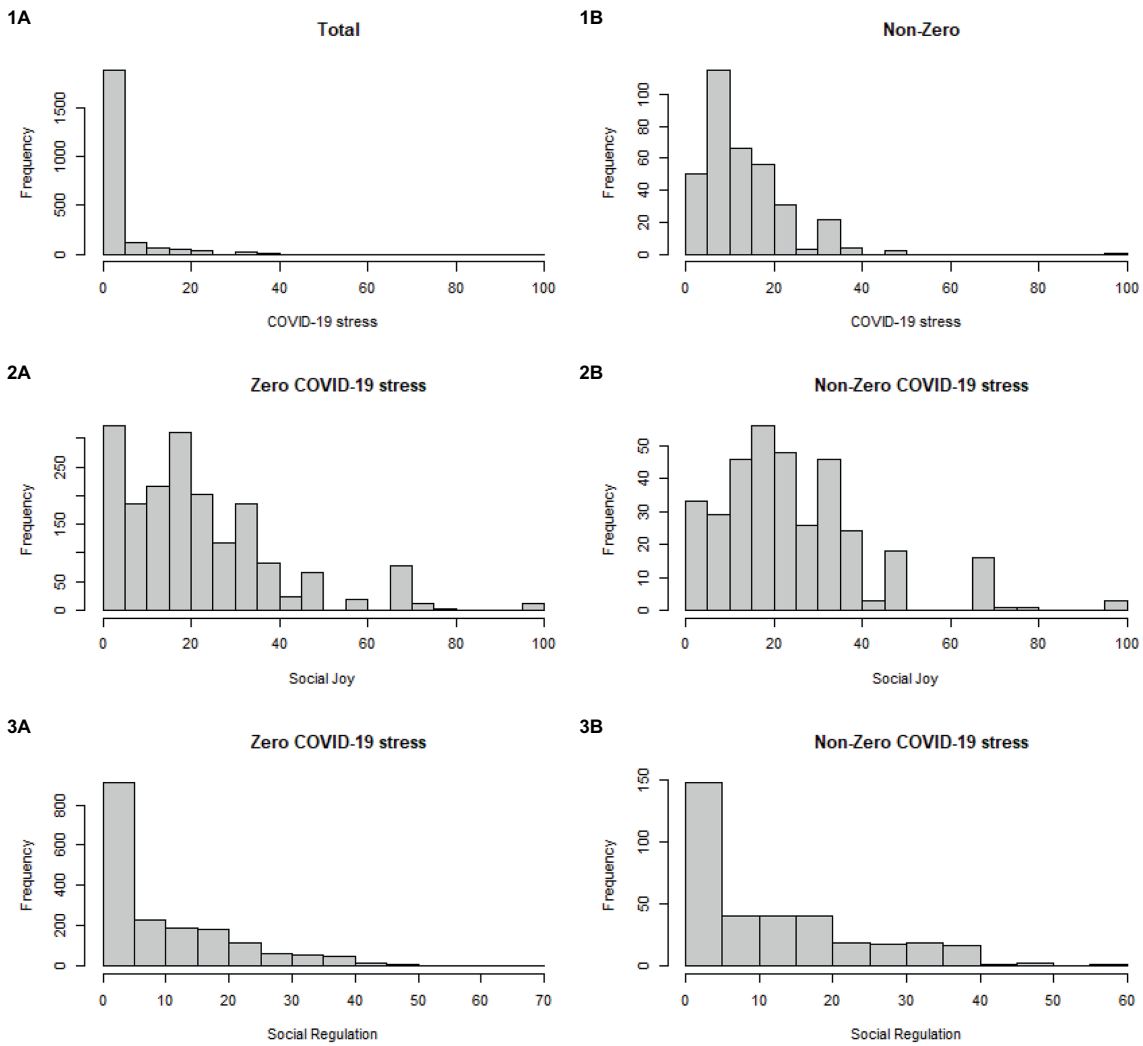
Frequency Distributions of Study Variables. **(1A)** shows the total frequency distribution of COVID-19 stress. **(1B)** shows the frequency distribution of COVID-19 stress with zeros excluded. **(2A)** shows the frequency distribution of Social Joy for individuals who did not report COVID-19 stress. **(2B)** shows the frequency distribution of Social Joy for individuals who did report COVID-19 stress. **(3A)** shows the frequency distribution for Social Regulation for individuals who did not report COVID-19 stress. **(3B)** shows the frequency distribution for Social Regulations for individuals who did report COVID-19 stress.

When we examined the prevalence of COVID-19 stress, we found many individuals did not write COVID-19 as a top 3 source of stress, and 86% of our responses were coded a zero (i.e., 86% of people did not indicate COVID-19 as their top 3 stressor; for frequency distributions of the study variables, see Figure 1). This was expected since participants need to mention key words related to COVID-19 to be coded, and we were only coding for top three stressors. We note that this is a limitation of our data which we address below by using analyses which can account for zero-inflation in our dependent variable. However, 14% participants still indicated COVID-19 as their top stressors without priming.

### Analyses Strategy

In order to deal with the zero-inflation of our dependent variable (i.e., many cases being coded as not finding COVID-19 as a top 3 stressor), we used a Tobit model regression, which is designed to handle truncated data with maximum likelihood estimation (McDonald and Moffitt, 1980). We entered our variables into a Tobit regression model in three steps.

In step one, we tested all demographic variables (race, gender, sexual orientation) simultaneously. In step two, we added the main effects of social joy and social regulation. Finally, in step three, we added the interaction of social joy and social regulation in order to see whether people who prefer both social joy and social regulation would experience even more COVID-19 stress. We examined the significance of our model coefficients at each step and we also examined the change in model fit (Pseudo *R*^2^ and Δ*χ*^2^).

## Results

Descriptive statistics and correlations between study variables were reported in Table 2. Spearman’s rank correlation coefficient is used because of the zero-inflation in COVID-19 stress. Initial analyses revealed sociability was positively correlated with COVID-19 stress. Specifically, finding joy in social interactions was positively correlated with appraising COVID-19 as a top stressor (*ρ* = 0.09, *p* < 0.001). Results were similar for the relationship between social regulation and COVID-19 stress. We found that individuals who preferred social regulation tended to appraise COVID-19 as more stressful (*ρ* = 0.07, *p* = 0.001). In addition, we found that COVID-19 stress was not significantly related to demographic group membership,^1^ including being a person of color (*F* (1, 2,181) = 0.20, *p* = 0.66), queer (*F* (1, 1957) = 0.21, *p* = 0.65), or female (*F* (1, 2,137) = 1.13, *p* = 0.29).

**Table 2:** Spearman’s rank correlations matrix.

	*Mean*	*sd*	1	2	3	4	5
COVID-19 stress (%)	2.21	6.52					
race	/	/	−0.02				
gender	/	/	0.02	0.00			
sexual orientation	/	/	0.01	−0.01	−0.05^*^		
social joy (%)	22.53	18.40	0.09^**^	0.03	0.01	−0.07^**^	
social regulation (%)	9.92	12.05	0.07^**^	0.01	0.06^**^	−0.01	0.10^**^

* *p* < 0.05;

** *p* < 0.01.

To account for the zero-inflated nature of our data, we chose to run all study variables in a hierarchical Tobit regression. The hierarchical regression model can be found in Table 3. In step one we entered our demographic variables (race, sexual orientation, and gender), none of which were significantly related to COVID-19 stress. In step two, both social joy (*β* = 2.62, *p* = 0.001) and social regulation (*β* = 2.49, *p* = 0.002) were significantly related to COVID-19 stress, and the addition of these effects improved model fit (Δ*χ*^2^ (2, *N* = 1934) = 21.53, *p* < 0.001). In step three, we did not find a significant interaction between social joy and social regulation use (*β* = −0.42, *p* = 0.76).

**Table 3:** Tobit regression coefficients.

COVID-19 stress	*β*	*SE*	Pseudo *R*^2^	Δ*χ*^2^ ^1^
**Step1**				
Race	−0.13	0.86		
Gender	1.15	0.88		
Sex Orientation	0.52	0.86		
			0.12	
**Step2**				
Race	−0.28	0.85		
Gender	1.12	0.87		
Sex Orientation	0.80	0.85		
Social Joy	2.62^**^	0.81		
Social Regulation	2.49^**^	0.82		
			0.13	21.53^*^
**Step3**				
Race	−0.27	0.85		
Gender	1.11	0.87		
Sex Orientation	0.79	0.86		
Social Joy	2.83^**^	1.05		
Social Regulation	2.80^*^	1.31		
Social Joy^*^Social Regulation	−0.42	1.42		
			0.13	0.09

* *p* < 0.05;

** *p* < 0.01.

1 Δ*χ*^2^ is calculated on the change of log likelihood.

## Discussion

In this study, we investigated sociability as an antecedent of experiences of stress early in the COVID-19 pandemic and explored if there were differences in experiences of stress based on demographic membership among preservice educators. Despite public and scientific interest in demographic group membership as a risk factor of COVID-19 stress, we did not find support for gender, sexual orientation, or race being associated with COVID-19 stress. We did, however, find that differences in sociability (operationalized as deriving joy from social interaction and using social support regulation strategies) did predict COVID-19 stress. In the section below, we discuss the theoretical and practical implication of our findings.

First, our study suggests that there were individual differences in the experience of the pandemic as those who find social interaction more important are deprived of it. The COVID-19 pandemic has dramatically altered norms governing social interaction and created a need to practice long-term social distancing and social isolation. Our findings suggest these pandemic practices may be appraised as more stressful by individuals who are high on sociability compared to individuals that prefer solitude. This suggests that people who are higher on sociability are more vulnerable to COVID-19 stress. Prior scholars have noted concerns about the impact of social isolation of COVID-19 on loneliness (Ozcelik et al., 2020; Way et al., 2021). Furthermore, research in general has also indicated a general rise in loneliness over time (Cigna, 2020). COVID-19 isolation could make loneliness more severe, especially for those who are high on sociability. Future study can further examine the relationship between COVID-19 loneliness and sociability.

Second, our research provides preliminary evidence and suggests more attention is needed to unpack the importance of social regulation. Research shows that people are not passively dealing with COVID-19 stress but rather the more stress they report, the more likely they have tried different types of strategies (Taylor et al., 2020a). During the pandemic, people spent more time on social media, games, TV shows, or other household activities in order to cope with the stress of the quarantine requirements (Ahmed et al., 2021). Seeking social support is a key emotion regulation strategy especially for people with high sociability (Vollrath et al., 1995), yet it is inhibited by the pandemic. Our findings suggest that COVID-19 stress may prove more difficult to deal with for people that rely social coping. Future research should explore what alternative strategies would be most effective in helping sociable people deal with their stress under the conditions of social isolation imposed by the pandemic. Because COVID-19 may constrain social regulation strategies, people who are high on sociability may benefit from other non-social forms of regulation or coping such meditation and mindfulness (Yalçın et al., 2021).

Finally, future research should explore the relationship between culture and COVID-19 stress. Although social interaction is necessary for people to maintain mental health, not all cultures favor seeking social support when dealing with stressors (Lay et al., 2020). For example, East Asian cultures discourage people from expressing negative emotions to friends and family in order to maintain relationship harmony (Matsumoto, 1996). People in collectivistic cultures are also willing to sacrifice their own personal joy for the greater good of the group (Triandis, 2018). This suggests that people in collectivistic cultures may be more willing to limit their own social interaction during the pandemic and may even derive joy or contentment from engaging in socially beneficial self-isolation. In fact, some studies have shown that people in more collectivistic cultures are more likely to wear masks (Lu et al., 2021). It may be the case that people from Eastern cultures which emphasize collectivism may experience less COVID-19 stress from isolation because collectivistic norms cause them to appraise quarantine and social distancing as maintaining social harmony. To date there is very little direct study of the interaction between culture and pandemic stress, and only one study has examined the effect of country-level individualism (Kowal et al., 2020). Future research should further explore the potential interactions between pandemic stress, culture, and sociability.

A limitation of our study is that our sample was not representative of the overall population of the United States. More than 75% of our sample were women and 100% belonged to a single occupation (educator). Thus, the interpretation of our data is limited by the generalizability of our sample. A second limitation of this study is the cross-sectional design which did not allow us to test the causal effects or the long-term effects of pandemic-related social isolation on sociable individuals. During the data collection period, the pandemic was in its early stages and most severe state which was characterized by uncertainty (including heightened social distancing and quarantine requirements, and the transition to remote work). Future studies should use longitudinal designs to explore the time lagged effects of deriving joy from social interaction and pandemic stress. A third limitation is the open-ended questions we used to code COVID-19. Many people did not write in COVID-19 as one of their top 3 stressors which means that our variable was non-normal. This may contribute to the small effect size in our study. Future research should consider using measures that have been developed to assess COVID-19 stress (e.g., COVID stress scales, see in Taylor et al., 2020a). Due to the data collection timing of our study, however, these measures were not yet available. Future studies could verify our results using other measures of COVID-19 stress. Finally, exposure to COVID-19 or having someone close get infected with COVID-19 may influence people’s experience and stress toward COVID-19 (Taylor et al., 2020b). Future research can explore how sociable people might be differentially affected by having these kinds of exposures to COVID-19.

## Data Availability Statement

The data supporting the conclusions of this article will be available at: https://osf.io/huj6s/.

## Ethics Statement

The studies involving human participants were reviewed and approved by Yale School of Medicine IRB. The patients/participants provided their written informed consent to participate in this study.

## Author Contributions

PL: conceptualization, methodology, formal analysis, investigation, and writing – original draft. ML: conceptualization, writing – original draft, and supervision. CC: writing – review and editing and project administration. MB: writing – review and editing and funding acquisition. All authors contributed to the article and approved the submitted version.

## Funding

The funding is provided by Teach for America, funding number YD000223.

## Conflict of Interest

The authors declare that the research was conducted in the absence of any commercial or financial relationships that could be construed as a potential conflict of interest.

## Publisher’s Note

All claims expressed in this article are solely those of the authors and do not necessarily represent those of their affiliated organizations, or those of the publisher, the editors and the reviewers. Any product that may be evaluated in this article, or claim that may be made by its manufacturer, is not guaranteed or endorsed by the publisher.

## Footnotes

^1^Because some demographic sub-groups have small samples and may be similar to one another on COVID stress, we decided to collapse these into broad demographic categories (e.g., being white vs. being a person of color, being straight vs. being queer). To determine the appropriateness of the collapsing, we first ran ANOVAs to compare differences among racial groups and sexual orientation groups. When we compared racial sub-groups on COVID-19 stress, we did not find any significant differences (*F* (5, 2003) = 1.28, *p* = 0.27). When we compared sexual orientation groups, no difference was found except for lesbians compared to some other groups (*F* (5, 2,177) = 2.55 *p* = 0.03); post-hoc Tukey tests revealed that lesbians had significant higher COVID-19 stress compared to straight (*p* = 0.01), gay (*p* = 0.01), bisexual (*p* = 0.02), and not answered/questioning/other (*p* = 0.01). However, we note that this finding is likely due to the small sample size of the lesbian group (n = 35). Given that we did not find substantive differences, we decide to collapse race into White vs. Person of Color, and sexual orientation into Straight vs. Queer.

## Appendix A

**Table:** *COVID-19 Stress in Subgroups*.

	*Mean*	*SD*	*N*
Male	1.95	5.79	500
Female	2.31	6.79	1,639
			
Bisexual	2.18	6.05	188
Gay	1.47	4.67	59
Lesbian	6.02	11.34	35
Queer	2.31	5.56	80
Straight	2.18	6.63	1,552
Not answered/Questioning/Other	2.16	6.00	269
			
African American	1.94	6.28	352
Asian	2.11	6.14	162
Hispanic	2.61	6.74	158
Multiracial	1.43	4.87	176
Native/Indigenous	4.32	7.22	16
White	2.25	6.80	1,070
Other of Color	2.76	6.45	37
